# In Vitro Biotransformation and Anti-Inflammatory Activity of Constituents and Metabolites of *Filipendula ulmaria*

**DOI:** 10.3390/pharmaceutics15041291

**Published:** 2023-04-20

**Authors:** Anastasia Van der Auwera, Laura Peeters, Kenn Foubert, Stefano Piazza, Wim Vanden Berghe, Nina Hermans, Luc Pieters

**Affiliations:** 1 Natural Products & Food Research and Analysis (NatuRA), Department of Pharmaceutical Sciences, University of Antwerp, Universiteitsplein 1, 2610 Antwerp, Belgium; anastasia.vanderauwera@uantwerpen.be (A.V.d.A.); laura.peeters@uantwerpen.be (L.P.); kenn.foubert@uantwerpen.be (K.F.); nina.hermans@uantwerpen.be (N.H.); 2 Laboratory of Pharmacognosy, Department of Pharmacological and Biomolecular Sciences, University of Milan, 20134 Milan, Italy; stefano.piazza@unimi.it; 3 Laboratory of Protein Chemistry, Proteomics & Epigenetic Signaling (PPES), Department of Biomedical Sciences, University of Antwerp, Universiteitsplein 1, 2610 Antwerp, Belgium; wim.vandenberghe@uantwerpen.be

**Keywords:** *Filipendula ulmaria*, Rosaceae, meadowsweet, gastrointestinal biotransformation, anti-inflammatory activity

## Abstract

(1) Background: *Filipendula ulmaria* (L.) Maxim. (Rosaceae) (meadowsweet) is widely used in phytotherapy against inflammatory diseases. However, its active constituents are not exactly known. Moreover, it contains many constituents, such as flavonoid glycosides, which are not absorbed, but metabolized in the colon by gut microbiota, producing potentially active metabolites that can be absorbed. The aim of this study was to characterize the active constituents or metabolites. (2) Methods: A *F. ulmaria* extract was processed in an in vitro gastrointestinal biotransformation model, and the metabolites were characterized using UHPLC-ESI-QTOF-MS analysis. In vitro anti-inflammatory activity was evaluated by testing the inhibition of NF-κB activation, COX-1 and COX-2 enzyme inhibition. (3) Results: The simulation of gastrointestinal biotransformation showed a decrease in the relative abundance of glycosylated flavonoids such as rutin, spiraeoside and isoquercitrin in the colon compartment, and an increase in aglycons such as quercetin, apigenin, naringenin and kaempferol. The genuine as well as the metabolized extract showed a better inhibition of the COX-1 enzyme as compared to COX-2. A mix of aglycons present after biotransformation showed a significant inhibition of COX-1. (4) Conclusions: The anti-inflammatory activity of *F. ulmaria* may be explained by an additive or synergistic effect of genuine constituents and metabolites.

## 1. Introduction

Preparations from the herb and/or flowers of *Filipendula ulmaria* (L.) Maxim. (Rosaceae) (meadowsweet) have been used traditionally since the late 16th and 17th centuries for the treatment of inflammatory diseases, as a diuretic and antirheumatic. In some countries of the European Union, tinctures or tincture-based products containing alcoholic extracts of Filipendulae Herba are on the market as food supplements used for complaints such as rheumatic and arthritic pain [1]. Moreover, its anti-inflammatory activity has been documented through in vitro immunomodulatory studies of roots, herb and flower extracts; the effect of methanolic extracts (0–250 μg/mL) from the aerial parts on different markers of inflammation such as antioxidant capacity, leukocyte ROS production, the COX-2/PGE2 pathway and cytokine secretions; and in vivo animal studies, including a rat model of inflammation induced via intraplantar injection of carrageenan (flower infusion), and a model with cisplatin-induced liver and kidney oxidative stress in rats (methanol extract of aerial parts). This supports the use of meadowsweet preparations against inflammatory diseases. However, the active constituents are not exactly known [2,3,4,5].

Recently, we have reported a generic and comprehensive extraction protocol and the phytochemical composition of this plant species, analyzed using UPLC-DAD-HRMS [6]. A rich diversity of phenolic constituents was (tentatively) identified. Moreover, several state-of-the-art comprehensive extraction protocols for untargeted plant metabolomics were thoroughly evaluated and compared [7]. Similar to *Salix* species, meadowsweet contains salicylates and its precursors (isosalicin and monotropitin), as well as other phenolic constituents. It has been shown to contain flavonoid aglycons (e.g., quercetin and kaempferol), glycosylated flavonoids (such as rutin, hyperoside, quercitrin, avicularin and astragalin) and hydrolysable tannins (tellimagrandin I and II, rugosin A, B, D, and E). Many studies overlook the poor absorption and extensive metabolism of polyphenols and their glycosides after oral intake, although it is a key factor for their bioactivity. A significant proportion of these constituents will reach the colon, and will be metabolized by the colonic microbiota, transforming these compounds to more absorbable molecules with potential bioactivity [6,8].

Therefore, an *F. ulmaria* extract was processed in an in vitro gastrointestinal biotransformation model [9]; the samples analyzed with UHPLC-ESI-QTOF-MS, and the data processed using a previously developed in-house workflow [10,11,12,13]. In addition, the anti-inflammatory activity of *F. ulmaria* and its possible metabolites on several key mediators in the inflammatory process was examined. Inhibition of the cyclooxygenase-1 and -2 (COX-1 and COX-2) enzymes was evaluated, because of the possible involvement of salicylates, known to act on these targets, in the biological activity of *F. ulmaria* extracts, as well as inhibition of activation of nuclear factor κB (NF-κB), a master switch in the process of inflammation. NF-κB regulates multiple genes involved in chronic inflammation by managing the transcription of inflammatory proteins such as chemokines, cytokines, interleukins, interferons and COX-2 [14]. Cyclooxygenase is the rate-limiting enzyme that plays a pivotal role in the conversion of arachidonic acid (AA) to the prostaglandin (PG) precursor PGH2. Non-steroidal anti-inflammatory drugs (NSAIDs) block the biosynthesis of prostaglandins, which mediate the typical signs of inflammation, through the inhibition of COX enzymes. COX-1 is expressed constitutively in many cells and tissues, synthesizing prostanoids involved in homeostasis and normal physiological functioning. It is also a valuable target for cardiovascular risk prevention, and certain types of cancer [15]. COX-2, on the other hand, has negligible basal expression, and can be induced strongly and rapidly [16]. The general aim of this work was to investigate, in an integrated way, how natural pro-drugs can be metabolized to active substances; this strategy was specifically applied to characterize anti-inflammatory compounds derived from *F. ulmaria*.

## 2. Materials and Methods

### 2.1. Chemicals

For the biotransformation experiments, ultrapure water with a resistivity of 18.2 MΩ·cm at 25 °C was generated with a Millipore™-purification system. UHPLC-grade MeOH, acetonitrile and formic acid were purchased from Biosolve (Dieuze, France), and dichloromethane from Merck (Darmstadt, Germany). The following analytical standards were provided by Sigma–Aldrich (St. Louis, MO, USA): apigenin, benzoic acid, caffeic acid, +/− catechin, chlorogenic acid, cinnamic acid, coumarin, emodin, epicatechin, ferulic acid, isorhamnetin, naringenin, *p*-coumaric acid, protocatechuic acid, quercetin, quercitrin, rutin, salicylic acid, sinapic acid, β-sitosterol, stigmasterol, syringic acid, tannic acid, taxifolin and vanillic acid. Luteolin and procyanidin B2 were provided by Santa Cruz Biotechnology (Santa Cruz, CA, USA). Gallic acid and p-hydroxybenzoic acid were provided by Carl Roth (Karlsruhe, Germany). All other chemicals and biochemicals were purchased from Sigma-Aldrich (St. Louis, MO, USA).

Reagents for the in vitro COX-1 and -2 enzyme inhibition assays were purchased as follows: DMSO (Uvasol), formic acid, TRIS/HCl, epinephrine hydrogen tartrate, porcine hematin and celecoxib from Sigma-Aldrich (St. Louis, MO, USA). Indomethacin was purchased from MP Biomedicals (Huissen, The Netherlands). Na2EDTA (Titriplex III) was purchased from VWR International (Leuven, Belgium). Arachidonic acid, purified COX-1 from ram seminal vesicles and human recombinant COX-2 are from Cayman Chemical (Ann Arbor, MI, USA). The competitive PGE2 EIA kit was purchased from Enzo Life Science (Farmingdale, NY, USA).

Murine fibrosarcoma L929 cells were purchased from ATCC (Manassas, VA, USA) and transfected with a TNF-induced NF-κB driven luciferase reporter gene construct by the lab of Protein Chemistry, Proteomics and Epigenetic Signaling (PPES) at the University of Antwerp. Dulbecco’s modified eagle medium (DMEM), fetal bovine serum (FBS), phosphate-buffered saline (PBS), penicillin, streptomycin and TNF- α (specific activity: 5.0 × 10^7^–2.0 × 10^8^ units/mg) were purchased from Gibco (Grand Island, NY, USA). DMSO (Uvasol) and dexamethasone were provided by Sigma-Aldrich (St. Louis, MO, USA). The luciferase assay system with reporter lysis buffer was purchased from Promega (Madison, WI, USA).

### 2.2. Preparation of Extract

Filipendulae Ulmariae Herba (batch number 19969), consisting of cut and dried flowering tops, was bought from Tilman SA (Baillonville, Belgium), and complied with the quality requirements of the European Pharmacopoeia. The dried plant material was ground prior to extraction with a PF 10 basic Microfine grinder drive (IKA-Werke GmbH & Co. KG, Staufen, Germany) using a sieve mesh size of 0.5 mm. To cover the full range of constituents in the plant material, a comprehensive extraction protocol with minor adaptations was applied, previously described by Bijttebier et al. [6]. The detailed protocol can be found in Supplementary Materials.

### 2.3. Preparation of Standard Solutions

Standard stock solutions of the analytical standards were prepared at a concentration of 1 mg/mL in UHPLC-grade MeOH for each analyte separately and stored in the dark at −80 °C. Dilutions of these solutions were prepared in 60:40 (v:v) MeOH:ammonium formate buffer (40 mM, aqueous). Standard stock and working solutions were stored at −80 °C in the dark.

### 2.4. Gastrointestinal Biotransformation

An in vitro gastrointestinal biotransformation model, previously developed and validated in-house, was used to mimic human biotransformation processes in the stomach, small intestine and colon including fermentation caused by pooled human feces. The digestive juices and fecal suspension were formulated to simulate the human conditions, which were previously described by Breynaert et al. [9] and Peeters et al. [11,12,13]. A brief overview of the protocol is described in Supplementary Materials.

This biotransformation experiment included three groups: samples containing the polar *F. ulmaria* extract (treated with digestive enzymes and fecal microflora (FEX)), negative control samples (also containing the extract with addition of digestive enzymes but not of fecal slurry (NCFEX)) and method blanks (containing no extract but comprising an equal volume of solvent and undergoing treatment with digestive enzymes and fecal bacteria (MB)). For preparation of the FEX samples, an amount of approximately 300 mg of the polar *F. ulmaria* extract was accurately weighed in triplicate and mixed with 47 mL of ultrapure water. In the same manner, NCFEX samples were prepared in duplo (containing the same amount of *F. ulmaria* extract) and 3 MB samples comprising an equal volume of water.

Sample aliquots were taken at several time points during the experiment: before biotransformation (t_0_), after the gastric phase (S, 1 h), after the small intestinal phase (SI, 1.5 h) and during different time points of the colon phase (after 2, 4, 6, 10, 14, 18, 22, 24, 32, 40, 48 and 72 h). Samples were diluted with methanol (1:2) and centrifuged at 10,000 rpm for 10 min. The supernatant was diluted 10 times with MeOH:H_2_O (60:40) before analysis.

### 2.5. Instrumental Analysis

The qualitative UHPLC-UV-QTOF analysis of the obtained biotransformation samples had been previously described by Peeters et al. [11,12,13]. The detailed description can be found in Supplementary Materials.

### 2.6. Data Analysis

In order to process the complex and dynamic data of the biotransformation experiment, a novel workflow was implemented to render as much information as possible from the longitudinal LC-MS data and to select the most interesting time profiles. This data analysis workflow was previously developed and validated using hederacoside C as a model compound [10,11]. The data analysis workflow is described in Supplementary Materials.

### 2.7. In Vitro Pharmacological Assays

#### 2.7.1. Sample Preparation

All samples were dissolved in DMSO (Uvasol) and aliquots of the stock solution were stored at −20 °C. For the cell-based assay, stock solutions of the samples were diluted in DMEM supplemented with penicillin and streptomycin, in order not to exceed a maximal concentration of 0.1% DMSO in the final test solutions. After aspirating the old medium from the cells, the diluted samples were added to the wells and the plates were incubated for 1 h. Test samples included a non-biotransformed *F. ulmaria* extract (20, 50 and 100 µg/mL) and a mixed sample (i.e., mix) composed of aglycons present after 72 h of fermentation (end concentrations in the assay were 20 µM of gallic acid and salicylic acid, 6 µM of quercetin and 4 µM of syringic acid). Dexamethasone at a concentration of 1 µM was used as a positive control.

For the COX enzyme inhibition assay, a non-biotransformed extract of *F. ulmaria* was tested at final concentrations of 5, 10, 25, 50 and 100 μg/mL. The concentrations were selected for a comparison with previous works [2,4]. The mix also contained a test concentration of 20 µM of gallic acid and salicylic acid, 6 µM of quercetin and 4 µM of syringic acid. Lastly, a preliminary experiment was performed to purify a 72 h fermentation sample of the *F. ulmaria* extract (i.e., FEX 72 h), as well as a blank fermentation sample (i.e., containing no extract, but including fecal suspension) via a methanol, ethyl acetate and acetone extraction protocol. These extracts were tested at a concentration of 10, 25 and 50 µg/mL. Indomethacin (1.25 μM) and celecoxib (2.5 μM) were used as positive controls for COX-1 and COX-2, respectively. Solvent vehicle (DMSO) did not exceed 2.5% in the final test solutions.

#### 2.7.2. COX Enzyme Inhibition Assay

COX-1 and COX-2 inhibition assays were performed in 96-well plates with purified COX 1 from ram seminal vesicles and human recombinant COX-2. From each test sample, 10 μL stock solution was added to 180 µL of the incubation mixture containing 0.1 M TRIS/HCl buffer (pH 8.0), 5 μM hematin, 18 mM epinephrine hydrogen tartrate, 1 unit/mL of enzyme preparation and 50 μM Na_2_EDTA (only added in the COX-2 assay). This was followed by a preincubation step for 5 min at room temperature. Initiation of the reaction was started by adding 5 μM arachidonic acid and incubating at 37 °C for 20 min. The reaction was terminated by addition of 10 μL formic acid 10%. The concentration of PGE2, the main arachidonic acid metabolite in this reaction, was determined immediately after the assay using a competitive PGE2 EIA kit from Enzo Life Sciences according to the manufacturer’s protocol. In short, the kit uses a monoclonal antibody to PGE2 to bind, in a competitive manner, the PGE2 in the sample, standard or an alkaline phosphatase molecule which has PGE2 covalently attached to it. After a simultaneous incubation of 2 h at room temperature, the excess reagents were washed away and the substrate was added. After 1 h of incubation time, the enzyme reaction was stopped and the absorbance was measured at 405 nm using a Biotek Eon plate reader (Agilent Technologies, Santa Clara, CA, USA). Inhibition of COX refers to the reduction in PGE2 formation in comparison to a blank vehicle control run without inhibitor.

#### 2.7.3. NF-κB Luciferase Reporter Gene Assay

Murine fibrosarcoma L929 cells transfected with a TNF-induced NF-κB driven luciferase reporter gene construct were cultured in DMEM supplemented with 10% heat-inactivated FBS, penicillin (100 units/mL) and streptomycin (100 μg/mL) in a humidified incubator under a 5% CO_2_ atmosphere at 37 °C. The L929 cells were seeded in 24-well plates at a cell density of 100,000 cells/well and incubated for 24 h before testing. The seeded L929 cells were pre-incubated with test samples for 1 h (37 °C), subsequently followed by a stimulation of 10 ng/mL recombinant mouse TNF-α for an additional 3 h (37 °C). The reporter quantitation was carried out following the manufacturer’s protocol from Promega. To summarize: after aspirating the cell medium, the L929 cells were lysed with reporter lysis buffer, which requires one single freeze–thaw cycle to achieve complete lysis. Lysates were vortexed, centrifuged at 12,000× *g* for 15 s and stored at −80 °C until analysis. Reading was performed using the GloMax 96 Microplate Luminometer from Promega (Madison, WI, USA). In a black 96-well plate (Greiner Bio-One, Vilvoorde, Belgium), 20 µL of cell lysate was pipetted manually per well, followed by the automatic addition of 100 µL Luciferase Assay Reagent per well in the GloMax 96 Microplate Luminometer. The produced light was measured for a period of 10 s after a delay time of 2 s.

#### 2.7.4. Data Analysis

The data were expressed as mean ± standard deviation (SD). Statistical evaluation of the data was performed through one-way analysis of variance (ANOVA), followed by the Tukey test, using GraphPad Prism, version 8.4.3. The results were considered statistically significant at *p* < 0.05.

## 3. Results

### 3.1. Gastrointestinal Biotransformation

**Figure 1:**
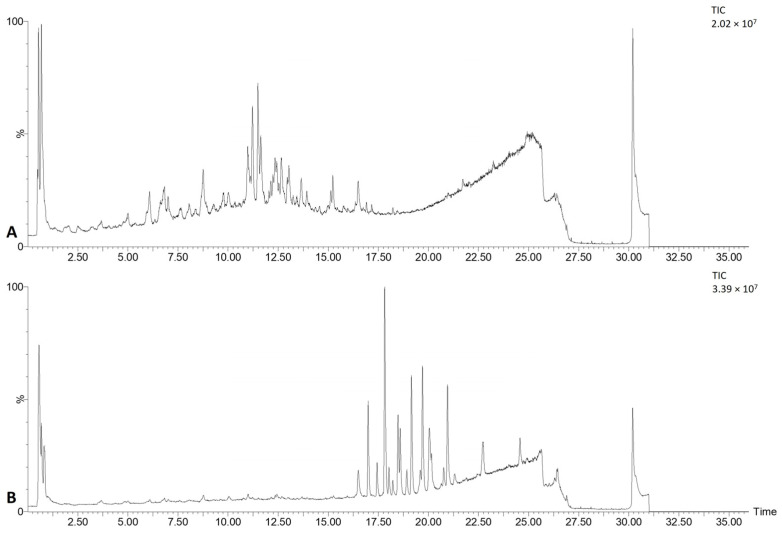
Total ion chromatogram of the *F. ulmaria* extract before (**A**) and after (**B**) in vitro gastrointestinal biotransformation.

Gastrointestinal biotransformation of the lyophilized *F. ulmaria* extract was simulated in vitro to monitor the level of the metabolites using am (accurate mass) MS and UV data. Figure 1 shows the chromatogram of the *F. ulmaria* extract before and after in vitro biotransformation. Before biotransformation (t_0_), peaks are mostly attributed to the tentatively identified compounds [6]. After the colonic phase of gastrointestinal biotransformation, the chromatogram contains peaks of genuine compounds, metabolites as well as matrix interferences, originating from enzymes, bile salts and fecal microflora, which are absent at t_0_.

The complexity of the data is thus increased immensely because of the presence of these gastrointestinal enzymes and fecal microflora in the samples, leading to a considerable amount of matrix-derived information. Moreover, the multiclass samples are measured as a function of time, adding a longitudinal aspect to the complex data and impeding the interpretation of the data. Manual processing of the chromatograms of every time point for every compound allows the monitoring of the intensity over time. The concentration of precursor compounds and metabolites can increase, decrease or show any combination of these patterns during biotransformation. However, this manual approach is very time-consuming and will only provide information about the abundance of previously identified compounds. Manual screening for metabolites is comparable to looking for a needle in a haystack because of the complexity of the chromatograms and matrix interferences. The automated data analysis workflow, previously described by Peeters et al. and Beirnaert et al., was thus used to screen rapidly and in an unbiased way for metabolites [10,11].

#### 3.1.1. Biotransformation of Flavonoid Glycosides

**Figure 2:**
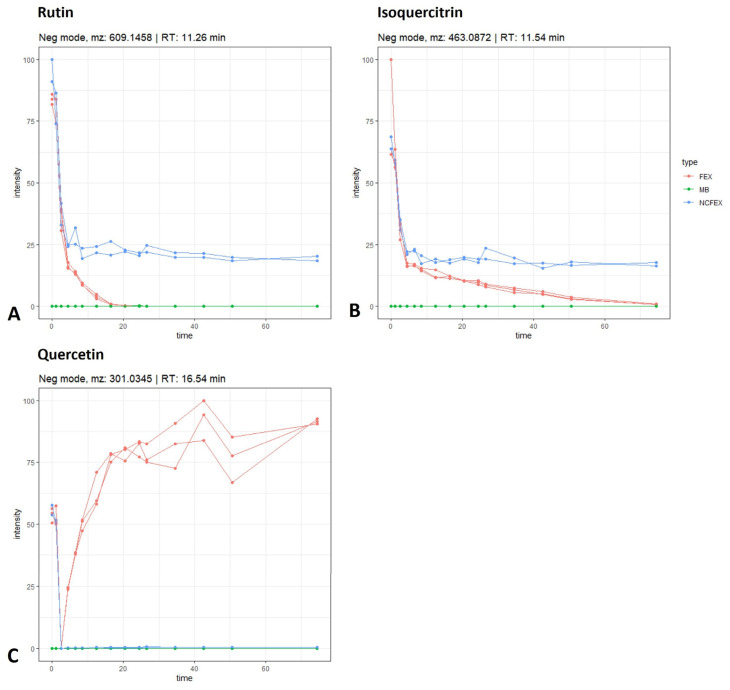
Time profiles (time in hours) of rutin (**A**), isoquercitrin (**B**) and quercetin (**C**) during gastrointestinal biotransformation. The gastric phase continued for 1 h, followed by the small intestinal phase for 1.5 h and a 72 h colonic phase. FEX (i.e., *F. ulmaria* extract) in red, NCFEX (i.e., negative control) in blue and MB (i.e., method blank) in green.

Quercetin glycosides, such as rutin, are abundantly present in *F. ulmaria* [6]. Rutin is a flavonol-*O*-glycoside composed of quercetin and rutinose, a disaccharide of rhamnose and glucose. The proposed biotransformation pathway of rutin in this experiment is summarized in Figure 2. A decrease in intensity of the molecular ion [M − H]^−^ of rutin (identified with a standard, *m*/*z* 609.1458; rt 11.26 min) can be observed over time. As shown in Figure 2A, this decrease is already detected in the stomach phase, and continues in the small intestinal phase and colonic phase until the signal is completely absent after 10 to 14 h of fermentation. In the negative control samples, due to the absence of fecal bacteria, the signal remains stable during the simulation of the colon phase. Lastly, the signal is not present in the method blank. The same pattern can be noticed for isoquercitrin (identified with standard, [M − H]^−^ *m*/*z* 463.0872; rt 11.54 min; Figure 2B). For quercetin (identified with standard, [M − H]^−^ *m*/*z* 301.0345; rt 16.54 min; Figure 2C), although already present at t_0_, a clear increase can be noted over time. When looking at the negative controls and method blanks, no increase in intensity can be observed. These results suggest the deglycosylation of rutin and/or isoquercitrin, leading to the formation of quercetin (see Figure 2). In the colon, the gut microbiota hydrolyze rutin, removing the sugar moiety and permitting absorption of the aglycone and/or extensive breakdown into low-molecular-weight phenolic metabolites [17,18].

**Figure 3:**
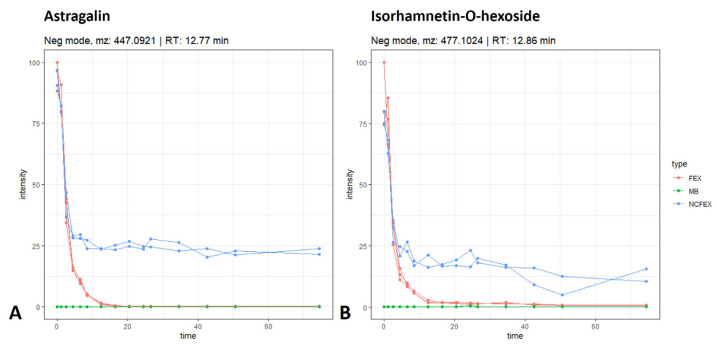
Time profiles (time in hours) of astragalin (**A**) and isorhamnetin-O-hexoside (**B**) during gastrointestinal biotransformation. See also legend of Figure 2.

However, quercetin can also be converted to other aglycons, such as kaempferol, luteolin and isorhamnetin (see below); nevertheless, these aglycones can also be formed from their corresponding glycosidic precursors. As an example, the time profile for astragalin (a kaempferol-*O*-glucoside) and isorhamnetin-*O*-hexoside is shown in Figure 3A,B, respectively. Astragalin was tentatively identified ([M − H]^−^ *m*/*z* 447.0921; rt 12.77 min) and can act as a precursor for kaempferol. Furthermore, isorhamnetin can be released from isorhamnetin-*O*-hexoside (tentatively identified, [M − H]^−^ *m*/*z* 477.1024; rt 12.86 min). Both glycosylated compounds follow a similar biotransformation time profile as rutin, isoquercitrin or spiraeoside: the negative control stays stable, the intensity in the biotransfomed samples decreases and the compounds are absent in the method blanks.

#### 3.1.2. Biotransformation of Flavonoid Aglycons

**Figure 4:**
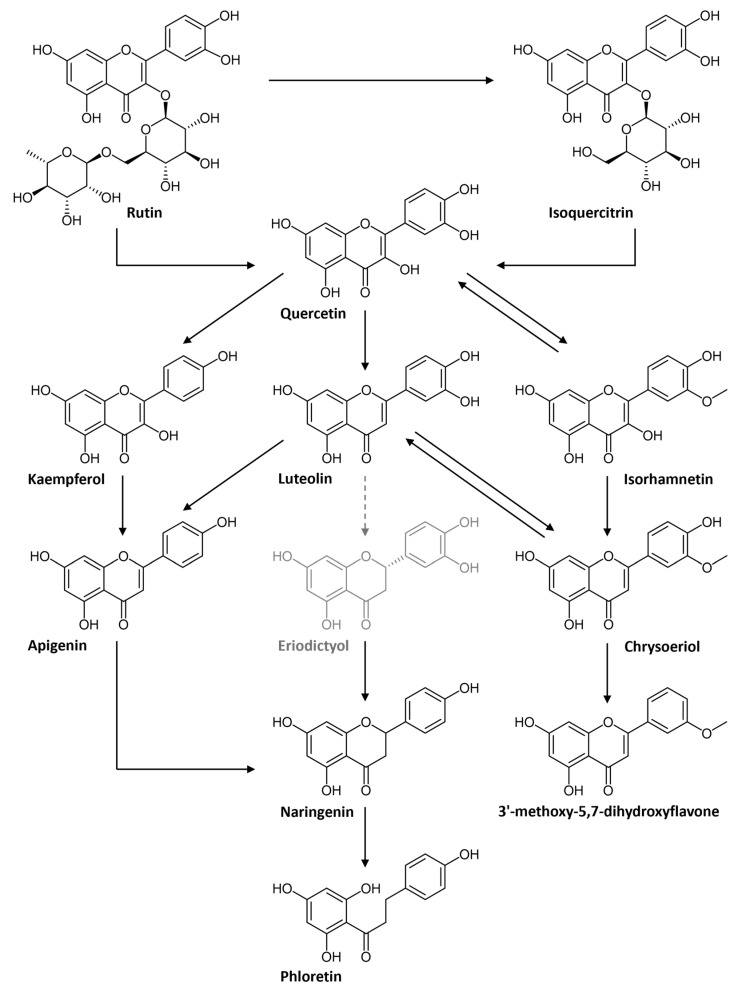
Metabolic pathway of rutin. Detected metabolites are in black; non-detected metabolites are in grey.

**Figure 5:**
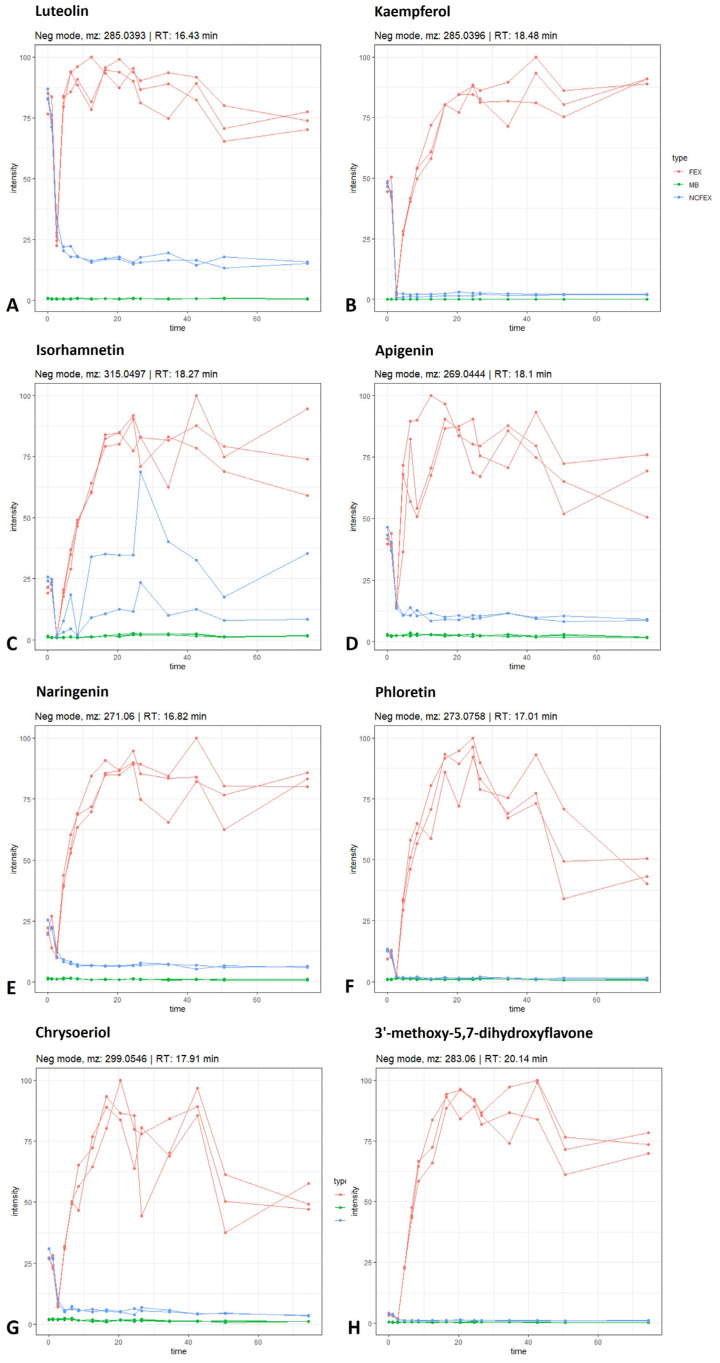
Time profile (time in hours) of luteolin (**A**), kaempferol (**B**), isorhamnetin (**C**), apigenin (**D**), naringenin (**E**), phloretin (**F**), chrysoeriol (**G**) and 3′-methoxy-5,7-dihydroxyflavone (**H**) during gastrointestinal biotransformation. See also legend of Figure 2.

The released quercetin aglycon can be further biotransformed by the intestinal microbiota (Figure 4). Quercetin can be dehydroxylated to kaempferol or luteolin, or can be converted to its metabolite isorhamnetin via catechol-*O*-methylation. An increase in kaempferol (identified with standard, [M − H]^−^ *m*/*z* 285.0396; rt 18.48 min), luteolin (identified with standard, [M − H]^−^ *m*/*z* 285.0393; rt 16.43 min) and isorhamnetin (identified with standard, [M − H]^−^ *m*/*z* 315.497; rt 18.27 min) is observed during colonic biotransformation, which differs from the negative control and method blank (Figure 5A–C). According to the scheme in Figure 4, it is possible for kaempferol and/or luteolin to be further dehydroxylated to apigenin ([M − H]^−^ *m*/*z* 269.0444; rt 18.10 min), which is indicated by the increasing intensity of apigenin in the fermented extract (Figure 5D). Furthermore, it is possible for apigenin to undergo reduction at the double bond between C-2 and C-3, resulting in the conversion to naringenin. On the other hand, reduction in luteolin at this double bond might lead to the formation of eriodictyol as an intermediate metabolite, eventually leading to the formation of naringenin via dehydroxylation. Although the metabolite eriodyctiol could not be detected in this in vitro experiment, an increasing intensity of naringenin (identified with standard, [M − H]^−^ *m*/*z* 271.0600; rt 16.82 min) could be observed (Figure 5E). Naringenin, in turn, is known as a precursor of phloretin (identified with standard, [M − H]^−^ *m*/*z* 273.0758; rt 17.01 min). The conversion of naringenin into naringenin chalcone is mediated by bacterial chalcone isomerase and the conversion of naringenin chalcone to phloretin by enoate reductase [19]. The increase in intensity of phloretin is shown in Figure 5F. With regard to isorhamnetin, it is possible that the *O*-methylated flavonol acts as a precursor for chrysoeriol (tentatively identified, [M − H]^−^ *m*/*z* 299.0546; rt 17.91 min). Moreover, luteolin can also be methylated at the 3′-position to generate chrysoeriol. Chrysoeriol, in turn, can be further dehydroxylated to a tentatively identified metabolite 3′-methoxy-5,7-dihydroxyflavone ([M − H]^−^ *m*/*z* 283.0600; rt 20.14 min). These biotransformation profiles are shown in Figure 5, where an increase in chrysoeriol (Figure 5G) and 3′-methoxy-5,7-dihydroxyflavone (Figure 5H) can be observed in the biotransformed samples over time.

#### 3.1.3. Biotransformation of Salicylic Acid Glycosides

**Figure 6:**
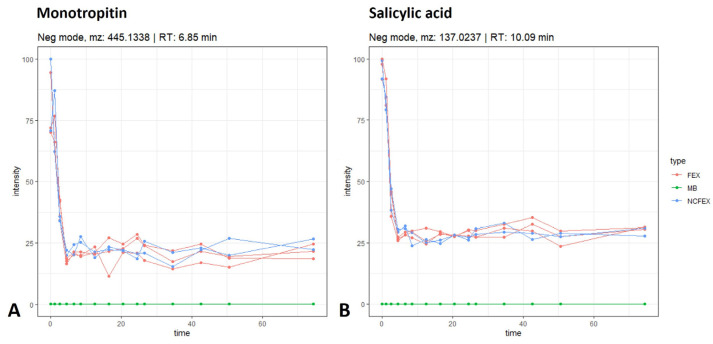
Time profiles (time in hours) of monotropitin (**A**) and salicylic acid (**B**) during gastrointestinal biotransformation. See also legend of Figure 2.

Glycosylated salicyl derivatives were detected in the *F. ulmaria* extract [6]. Figure 6 shows the time profile for the glycosylated compound, monotropitin (tentatively identified, *m*/*z* 445.1338; rt 6.85 min), and the aglycon salicylic acid (identified with standard, *m*/*z* 137.0237; rt 10.09 min). The biotransformation profile of both monotropitin and salicylic acid shows a drop in intensity, observed during the stomach and small intestinal phase in both the samples and the negative controls. In these phases, there is no difference between samples and negative controls. It is most likely that multiple minor metabolites are formed as the formation of one major metabolite was not observed in the stomach and small intestine. In the colon phase, however, no further changes in intensity were observed for monotropitin and salicylic acid. Additionally, no difference could be noted between the samples and the negative controls in the colon phase, indicating that there is no colonic biotransformation observed for these compounds in the samples.

#### 3.1.4. Biotransformation of Ellagitannins

**Figure 7:**
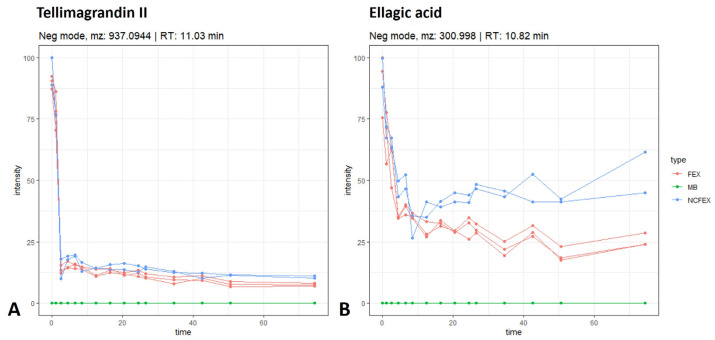
Time profiles (time in hours) of tellimagrandin II (**A**) and ellagic acid (**B**) during gastrointestinal biotransformation. See also legend of Figure 2.

In vivo and in vitro studies have shown that food products and medicinal plants rich in ellagitannins are able to be metabolized by gut microbiota to dibenzopyran-6-one derivatives with different hydroxyl substitutions, i.e., urolithins. In contrast to the ellagitannins present in ingested foods and extracts, urolithins possess good bioavailability and can be found in plasma at low micromolar concentrations [20,21]. Contrary to the results obtained by Piwowarski et al., in which an aqueous *F. ulmaria* extract was fermented, the formation of urolithins was not observed in our biotransformation experiments [21,22]. After the stomach and small intestinal phase, no further breakdown is observed for monomeric ellagitannins such as tellimagrandin I, tellimagrandin II, rugosin A, rugosin B, casuarinin, casuarictin and pedunculagin. A similar pattern was observed for the dimeric ellagitannins rugosin D and E. Figure 7A shows the biotransformation time profile for tellimagrandin II (tentatively identified, *m*/*z* 937.0944; rt 11.03 min) as an example. A decrease can be noted in the intensity of ellagic acid (tentatively identified, *m*/*z* 300.9980; rt 10.82 min (Figure 7B)). The decrease in ellagic acid, however, did not lead to the expected formation of any of the known urolithins (i.e., the precursor luteic acid, urolithin M5, M6, M7, A, B, C and isourolithin A or B), even after 72 h of fermentation.

### 3.2. Pharmacological Activity

#### 3.2.1. COX-1 and COX-2 Enzyme Inhibition

**Figure 8:**
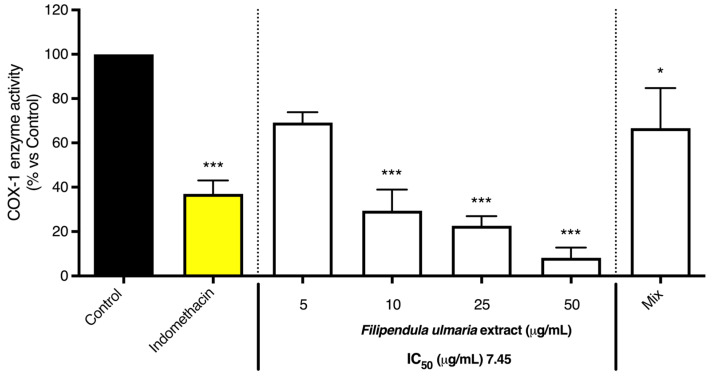
The effect of the *F. ulmaria* extract and the mix (20 µM of gallic acid and salicylic acid, 6 µM of quercetin and 4 µM of syringic acid) on COX-1 enzyme inhibition. Indomethacin (1.25 µM), in yellow, served as positive control. The graph depicts compiled data of three independent experiments (mean ± SD). *p*-values are expressed as * *p* < 0.05 and *** *p* < 0.0001 compared to control.

**Figure 9:**
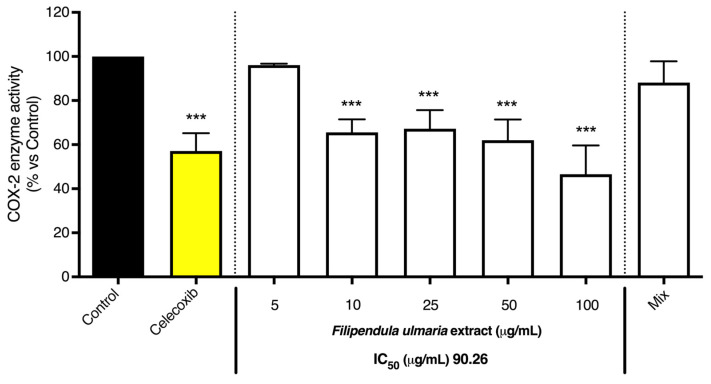
The effect of the *F. ulmaria* extract and the mix (20 µM of gallic acid and salicylic acid, 6 µM of quercetin and 4 µM of syringic acid) on COX-2 enzyme inhibition. Celecoxib (2.5 µM), in yellow, served as positive control. The graph depicts compiled data of three independent experiments (mean ± SD). *p*-values are expressed as *** *p* < 0.0001 compared to control.

As shown in Figure 8 and Figure 9, the non-biotransformed *F. ulmaria* extract inhibited COX-1 and -2 enzyme activities in a dose-dependent manner. *F. ulmaria* proved to be a more potent inhibitor of the COX-1 enzyme, with an IC_50_ of 7.45 µg/mL, compared to the results obtained in the COX-2 enzyme inhibition assay. For COX-2 enzyme inhibition, an IC_50_ of 90.26 µg/mL was achieved. Table 1 shows a summary of the IC_50_ on COX, with respective 95% C.I. (Table 1).

Several of the aglycons present in *F. ulmaria* after biotransformation were tested in the COX enzyme inhibition assay at a concentration of 20 µM (including quercetin, apigenin, luteolin, kaempferol, phloretin, salicylic acid, syringic acid and gallic acid). None of these isolated compounds appeared to significantly inhibit the COX-1 or -2 enzyme at the test concentration. To further link the presence of compounds and/or metabolites present after gastrointestinal biotransformation to pharmacological activity, a mix containing different groups of constituents present in the extract after biotransfomation was prepared, since testing samples containing fecal suspension proved to be difficult and challenging in cell-based assays such as the NF-κB luciferase reporter gene assay. Quercetin was used as a model compound for flavonol aglycons, and salicylic acid as a model compound for salicylates, the most abundant hydroxybenzoic acids in the herbal extract. Additionally, gallic acid was added as a possible metabolite of hydrolysable tannins and syringic acid as a product of microbial gut biotransformation of anthocyanins and other polyphenols. This mix thus contained a combination of 20 µM of gallic acid and salicylic acid, 6 µM of quercetin and 4 µM of syringic acid. Interestingly, the mix did show a significant inhibition (*p* < 0.05) of 33.33% of the COX-1 enzyme. This might be explained by a synergistic or additive effect, since the single compounds (all tested at a concentration of 20 µM) showed no clear activity on the enzyme. For the COX-2 enzyme, however, no significant activity was observed for the mix.

**Figure 10:**
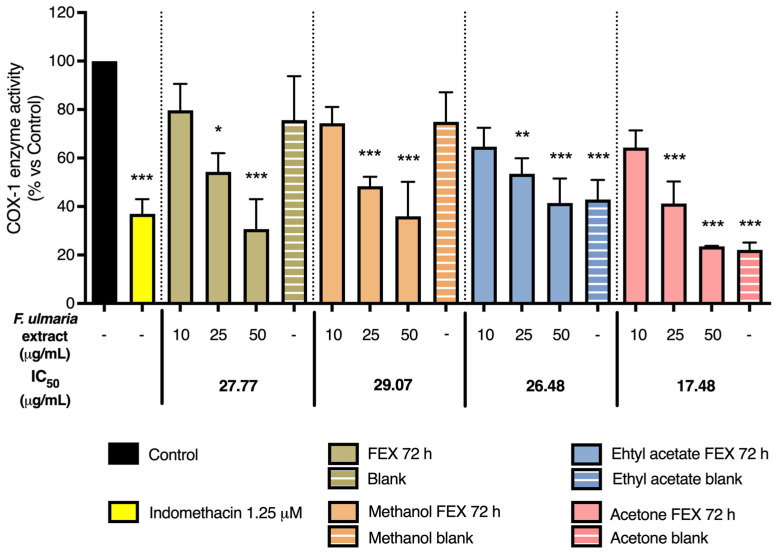
The effect of the digested *F. ulmaria* extract (FEX 72 h) and different extraction procedures of FEX 72 h on COX-1 enzyme inhibition. Indomethacin (1.25 µM), in yellow, served as positive control. The graph depicts compiled data of three independent experiments (mean ± SD). *p*-values are expressed as * *p* < 0.05, ** *p* < 0.01 and *** *p* < 0.0001 compared to control.

**Figure 11:**
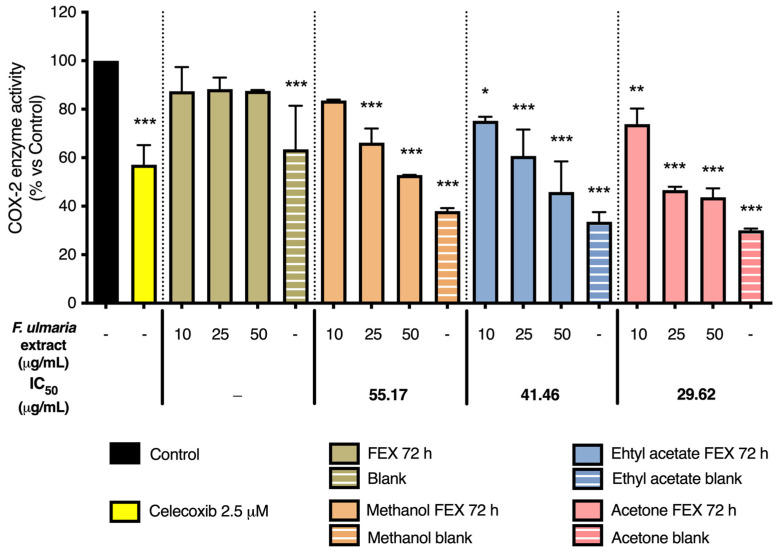
The effect of the digested *F. ulmaria extract* (FEX 72 h) and different extraction procedures of FEX 72 h on COX-2 enzyme inhibition. Celecoxib (2.5 µM), in yellow, served as positive control. The graph depicts compiled data of three independent experiments (mean ± SD). *p*-values are expressed as * *p* < 0.05, ** *p* < 0.01 and *** *p* < 0.0001 compared to control.

Finally, a preliminary experiment was performed in order to clean up the colon samples from the gastrointestinal model. Different extraction methods were applied on a 72 h fermentation sample of a *F. ulmaria* extract, named FEX 72 h, as well as a blank sample (including fermentation without addition of the extract). These results are summarized in Figure 10 and Figure 11. Firstly, the pure FEX 72 h sample was tested at a concentration of 10, 25 and 50 µM (in green). Secondly, three different extraction methods were compared in the same concentration range: a methanol extraction (in orange), an ethyl acetate extraction (in blue) and an acetone extraction (in pink). Across all different extraction methods and the pure FEX 72 h sample, a preference in selectivity for the COX-1 over the COX-2 enzyme can still be observed, which is similar to the results obtained for the non-biotransformed *F. ulmaria* extract. The IC_50_ values are summarized in Table 2: the acetone extraction showed the lowest values; polarity also does not seem to explain the trend observed for the different IC_50_ values. Thin-layer chromatography (TLC) confirmed the absence of flavonoids in the different blank samples. Surprisingly, a very clear effect can also be observed for the blank samples extracted with ethyl acetate or acetone, but not for the original blank sample or methanol blank.

#### 3.2.2. NF-κB Luciferase Reporter Gene Assay

**Figure 12:**
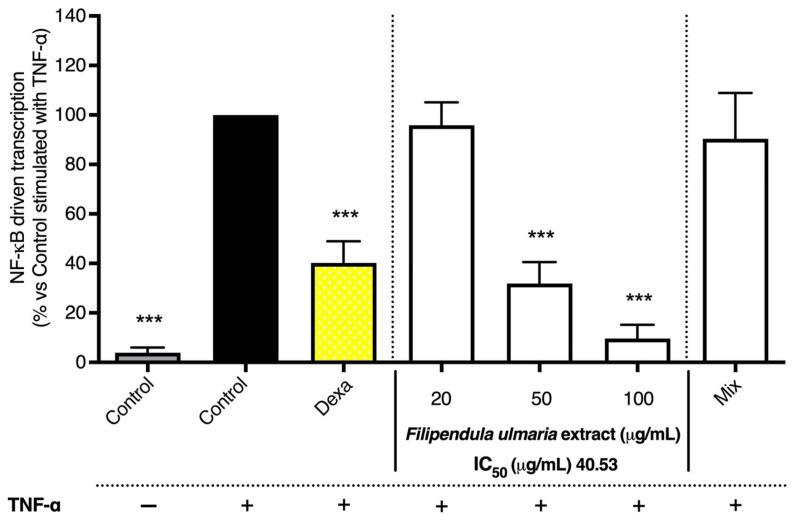
The effect of the *F. ulmaria* extract and the mix (20 µM of gallic acid and salicylic acid, 6 µM of quercetin and 4 µM of syringic acid) on NF-κB-driven transcription in L929 cells. Dexamethasone (Dexa, 1 µM), in yellow, served as positive control. The graph depicts compiled data of three independent experiments (mean ± SD). *p*-values are expressed as *** *p* < 0.0001 compared to control stimulated with TNF-α.

The comprehensive extract of *F. ulmaria* (i.e., the non-biotransformed extract) showed a clear inhibition on the NF-κB reporter gene induction by TNF-α in L929 cells, compared to the stimulated control, reaching an IC_50_ of 40.53 µg/mL, and also showed a clear dose–response behavior (see Figure 12). The mix, composed of stable aglycons present after 72 h of fermentation as described above, was also tested. However, for the mix, no significant activity was observed.

## 4. Discussion

An innovative strategy was used to disclose metabolic pathways, namely in vitro biotransformation via a gastrointestinal simulation model followed by metabolomics profiling. The gastrointestinal biotransformation pathway of several compounds present in an *F. ulmaria* extract was investigated using a non-targeted approach.

Quercetin glycosides, such as rutin, are abundantly present in *F. ulmaria*. When looking at the in vitro gastrointestinal biotransformation of rutin and/or isoquercitrin, deglycosylation is suggested as the likeliest biotransformation reaction, leading to the formation of quercetin. In the colon, the gut microbiota hydrolyze rutin, removing the sugar moiety and permitting absorption of the aglycon and/or extensive breakdown into low-molecular-weight phenolic metabolites such as phenylpropionic acid, phenylacetic acid and benzoic acid derivatives. Bacteria with the ability to biotransform rutin possess α-L-rhamnosidases which convert rutin into quercetin-3-glucoside (isoquercitrin). Bacteria can also express β-D-glucosidases that either convert quercetin-3-glucosides to quercetin or convert rutin directly to quercetin [23,24]. Lactobacillus acidophilus, Lactobacillus plantarum and Bifidobacterium dentium have been shown to have α-rhamnosidase activity involved in deglycosylation of flavonoids [25,26]. Bacteroides uniformis, Bacteroides ovatus and Enterococcus avium, on the other hand, have the capability of degrading rutin to quercetin [27,28]. Parabacteroides distasonis was shown to produce both quercetin-3-glucosides and quercetin via α-rhamnosidase and β-glucosidase activity [27]. Eubacterium ramulus and Enterococcus casseliflavus are able to biotransform quercetin-3-glucosides into quercetin [29,30]. Other glycosylated precursors of the quercetin aglycon, such as spiraeoside, quercitrin, avicularin and miquelianin, were also present in the *F. ulmaria* extract and followed similar gastrointestinal biotransformation time profiles, where a decrease in intensity was observed during gastrointestinal biotransformation.

Furthermore, a rise in intensity during the colon phase was also observed for a whole array of other aglycons such as kaempferol, luteolin, isorhamnetin, apigenin, naringenin, phloretin, chrysoeriol and a methoxyflavone. In the literature, it is described that only a small part of released quercetin aglycon is absorbed by colonocytes in its intact form. A large percentage of the released quercetin is absorbed after a series of biotransformation reactions into phenolic catabolites by the colonic microbiota [31]. Biochemical transformations by the gut microbiota include three major catabolic processes: hydrolysis (deglycosylations and ester hydrolysis), cleavage (C-ring cleavage, delactonization, demethylation) and reductions (dehydroxylation and double bond reduction) [32]. However, it is also possible that these compounds originate from their corresponding glycosylated precursor, e.g., kaempferol-O-glucoside and isorhamnetin-O-hexoside, and followed a similar biotransformation time profile as rutin, resulting in kaempferol and isorhamnetin, respectively.

When looking at precursors of salicylic acid, no further breakdown of monotropitin or increase in intensity for salicylic acid was observed in the colon phase. In the literature, it is described that after oral ingestion, salicin is hydrolyzed to its aglycon saligenin by the gut microbiota and saligenin is then further oxidized in the liver to salicylic acid. Early in vitro studies from Fötsch and Pfeifer, using intestinal sections of normal and antibiotic-treated rats, found that the intestinal bacteria were able to convert salicin to saligenin [33]. However, when comparing these results to in vivo pharmacokinetic studies in humans, the metabolite salicylic acid could already be detected in serum 1 h after oral administration of salicin or willow bark extract, which is rich in salicylates. This suggests that salicin might already be absorbed in the stomach or upper intestinal tract and hydrolyzed before or during absorption, thus even before reaching the colon [34]. In vivo research in rats carried out by Knuth et al. showed similar results when salicortin was orally administered [35]. In the study of Pferschy-Wenzig et al., where willow bark extract was incubated with human fecal suspension under anoxic conditions (without being preceded by a gastric and small intestinal phase), the formation of saligenin and salicylic acid was observed. Nevertheless, it was also mentioned that hydrolysis by intestinal bacteria might not be relevant under physiological conditions [36]. Moreover, bioavailability studies of pure salicin in humans resulted in salicylic acid metabolites, indicating a good oral bioavailability of the pure compound [37]. In contrast, oral administration of willow bark extract, which is rich in salicylates, showed lower bioavailability of salicin [34,38]. The expected (intermediate) metabolite saligenin could not be detected. This, however, might be attributed to the applied ESI-MS conditions, which might not allow the satisfactory detection of some analytes present in the samples. Small aromatic compounds such as saligenin, might ionize only weakly: saligenin could also not be detected in the mixture of reference compounds, used for MS analysis, thereby hampering the detection of this microbial metabolites. Application of a second analytical technique, such as GC-MS, might be a solution for future studies.

Although *F. ulmaria* is rich in ellagitannins, the expected urolithin metabolites were not detected in the biotransformation experiment. No further breakdown was observed in monomeric ellagitannins and in dimeric ellagitannins after the initial decline observed in the stomach and small intestinal phase. These slower or incomplete biotransformation rates could be caused by the presence of diverse compounds in the crude extract, which could negatively interfere with the biotransformation rate of some compounds. Another hypothesis is that biotransformation reactions are hampered by a possible antibacterial effect of the extract, resulting in an insufficient amount of viable bacteria or an altered bacterial composition, inhibiting or changing biotransformation. Furthermore, a large inter- and intraindividual variability in gut microbiota exists and is an important confounding factor in studies on the formation of effect of nutrients, drugs and phytochemicals. Metabotype A is characterized by the production of urolithin A, whereas metabotype B produces urolithin A, isourolithin A and urolithin B. However, metabotype 0 does not produce any of these final urolithins [39,40]. The genus Gordonibacter from the Eggerthellaceae family accommodates bacterial species, namely G. pamelaeae and G. urolithinfaciens that can biotransform ellagic acid into urolithin M5, M6 and C [41]. Ellagibacter isourolithinifaciens, another genus from the Eggerthellaceae family, can also convert ellagic acid into urolithin M5, M6, C and isourolithin A. Strains of the closest neighbors of Gordonibacter, i.e., Paraeggerthella and Eggerthella, and Ellagibacter, i.e., Senegalimassilia and Adlercreutzia, were tested for their ability to catabolize ellagic acid but were unable to produce urolithins [42].

As a last step, the activity was evaluated with in vitro anti-inflammatory assays, focusing on COX and NF-κB. It can be concluded that *F. ulmaria* clearly exerted anti-inflammatory potential, which confirms its use in European traditional medicine against inflammatory diseases. The non-biotransformed *F. ulmaria* extract showed a clear dose-dependent inhibition on the TNF-α-induced NF-κB luciferase reporter gene induction in L929 cells compared to the stimulated control. Additionally, the activity of both COX isoenzymes was also inhibited in a dose-dependent manner and showed to be a more potent inhibitor of the COX-1 enzyme compared to the COX-2 enzyme. These results are in line with literature [4,5,43,44].

While evidence from such in vitro experiments does not necessarily prove its in vivo activity, these studies do support and provide a rationale for the use of *F. ulmaria* to suppress inflammation in vivo. Interpreting data obtained in this way, however, requires caution. First of all, the bioactive compounds might be formed in vivo due to biotransformation reactions carried out by the intestinal microflora or by the hepatic metabolism, which means they were not present in the original extract. Secondly, another important aspect is whether the concentration of the extract’s constituents are realistic or achievable in vivo. Typically, many plant constituents are not completely bioavailable since they are not easily absorbed. Therefore, a preliminary experiment was performed in order to clean up the fermented *F. ulmaria* extract of the gastrointestinal model, using methanol, ethyl acetate and acetone extraction. A preference in selectivity for the COX-1 over the COX-2 enzyme could still be observed in the enzyme inhibition assay. Surprisingly, a very clear effect can also be observed for the blank samples extracted with ethyl acetate or acetone. Therefore, the observed inhibition could be due to other compounds present in these blank samples, such as short-chain fatty acids (SCFAs). Hence, the contribution of SCFAs would be an interesting lead to follow. SCFAs, primarily butyric, acetic or propionic acid, are the bacterial end products of fiber fermentation processes. García-Villalba et al., for example, found that liquid–liquid extraction with ethyl acetate showed good extraction efficiencies of SCFAs in aqueous fecal suspensions. There is a growing interest in these compounds due to increasing evidence of their positive physiological effects, especially in relation to colonic function, as they contribute to normal bowel function, colonic vasculature and musculature. Moreover, their absence has been associated with inflammatory bowel diseases and they seem to play an important role in the protection against colon cancer [45].

Testing samples containing fecal material proved to be difficult and challenging in cell-based assays; therefore, a mix composed of aglycons present after 72 h of fermentation (containing 20 µM of gallic acid and salicylic acid, 6 µM of quercetin and 4 µM of syringic acid) was used to test the effect on TNF-α-induced NF-κB luciferase reporter gene induction in L929 cells. It has been shown that polyphenols target multiple inflammatory mechanisms, one of them being the inhibition of the activation of NF-κB [46]. However, the results of the mix are not completely in line with the literature, which might be due to differences in test concentrations and assay conditions. For example, the suppression of TNF-α-induced NF-κB activation by gallic acid (IC_50_ of 76 µM) has been determined with stably transfected 293/NF-κB-Luc human embryonic kidney cells [47]. In a more recent paper from Lu et al., the NF-κB inhibitory effect of gallic acid (ranging from 1 to 100 µM) was also shown in transfected HEK293 cells [48]. Research has shown that salicylic acid inhibits the activation of NF-κB. However, these observations are based on in vitro research using high concentrations of aspirin and sodium salicylate, and their relevance should be questioned in an in vivo situation [49]. Kopp and Ghosh, for example, showed that sodium salicylate (and aspirin), ranging between 2 and 20 mM, was able to prevent the degradation of IκB in a dose-dependent manner [50]. Similar results were obtained in subsequent studies by Bayón et al. [51], Grilli et al. [52], Pierce et al. [53] and Yin et al. [54], testing salicylic acid in millimolar concentrations. Quercetin has also been reported to inhibit NF-κB activation. In an article from Cho et al., quercetin (1–20 µM) significantly inhibited NF-κB reporter gene activity in a dose-dependent manner in a luciferase reporter assay in HUVEC cells [55]. A recent article from Fang et al. showed an inhibiting effect of syringic acid on the expression of p65 and phosphorylated IκBα in an ulcerative colitis model mice induced with dextran sulfate sodium (orally treated with 25 mg/kg bodyweight syringic acid), as well as in LPS-induced RAW 264.7 macrophages (10–20 µM) [56].

Interestingly, the mix showed a significant inhibition on the COX-1 enzyme. This might be explained by a synergistic or additive effect, since the single compounds tested in the assay showed no clear activity on the enzyme. For the COX-2 enzyme, no significant activity was observed for the mix, as well as for the single compounds. Contrary to our results, in a study carried out by Al-Fayez et al., quercetin inhibited the enzyme activity in purified COX-1 and -2 preparations, reaching an IC_50_ near 5 µM [57]. El-Seedi et al. also found an inhibitory effect of quercetin on the COX-1 enzyme: an inhibition of 44% was achieved when testing at a relatively high concentration of 200 µM [58]. In an article from Kutil et al., phenolic compounds from wine were investigated for COX-1 and COX-2 inhibitory activity. However, weak activity was found for quercetin, with an IC_50_ of 44 µM in the case of COX-1 [59]. Only weak inhibition of COX by salicylic acid has been found in several studies. In purified enzyme preparations, sodium salicylate was inactive up to 6.25 mM [60]. Experiments in intact cells also found salicylic acid to be a weak inhibitor of COX-1, with an IC_50_ that is usually more than 1.5 mM [61,62,63]. For COX-2 enzyme inhibition, it seems to depend on the experimental conditions (IC_50_ values ranging between 30 µM and 1.5 mM) [61,64]. Gallic acid, in contrast to our results, is known in literature to inhibit both COX isoenzymes. Madlener et al. determined IC_50_ values of 3.5 and 4.4 nM for COX-1 and COX-2, respectively [65]. Reddy et al. also found a strong inhibitory effect of gallic acid, with an IC_50_ of 1.5 µM for COX-1 and 74 nM for COX-2, thus showing a much stronger preference towards COX-2 [66]. In a cell-based experiment from the research group of Stanikunaite, a strong inhibition of the COX-2 enzyme (IC_50_ of 2 µM) in LPS-stimulated RAW 264.7 cells was observed for syringic acid. These results were determined by the conversion of exogenous arachidonic acid to PGE2 [67]. The overall data suggested that compounds already present in the original extract, potentially stable after gastrointestinal digestion, may account for the inhibitory effect on COX-1 enzyme. In fact, both the non-biotransformed *F. ulmaria* extract, the digested FEX 72 h and the mix of aglycons show a comparable effect in the COX-1 assay. On the contrary, the effect of *F. ulmaria* extract on COX-2 may have regarded both enzymatic and expression inhibition. The latter may involve NF-κB impairment. However, taking apart the notable matrix effect observed in this context, COX-2 enzymatic inhibition might be strongly affected by the biotransformation process.

To conclude, in vitro gastrointestinal biotransformation did not result in a single major metabolite responsible for the anti-inflammatory activity of *F. ulmaria*. As the extract shows activity on COX-1, an additive or synergistic effect of multiple minor compounds and/or their metabolites present in the extract of *F. ulmaria* is suggested.

## 5. Conclusions

The scope of this research was to develop an integrated strategy, based on natural pro-drugs and their metabolites, to characterize anti-inflammatory compounds derived from *F. ulmaria*. In this work, we clearly stated the fundamental role of colon biotransformation for the comprehension of the biological activity of complex plant extracts. To conclude, this new integrated approach offers added-value medicinal plant research, enabling the phytochemical identification of compounds and their metabolites after in vitro biotransformation, followed by preliminary in vitro activity testing, omitting time-consuming and expensive in vivo studies in the early stages of research.

## Figures and Tables

**Table 1:** IC_50_ of *F. ulmaria* extract on COX activity.

*F. ulmaria* Extract	IC_50_ (µg/mL)	95% C.I.
COX-1	7.45	5.46 to 9.83
COX-2	90.26	60.95 to 135.70

95% C.I.; 95% interval of confidence.

**Table 2:** IC_50_ of digested *F. ulmaria* extract on COX activity.

Digested *F. ulmaria* Extract	COX Isoform	IC_50_ (µg/mL)	95% C.I.
FEX (72 h)	COX-1	27.77	18.39 to 38.51
Methanol FEX		29.07	19.14 to 44.14
Ethyl acetate FEX		26.48	19.33 to 36.30
Acetone FEX		17.48	13.09 to 23.34
FEX (72 h)	COX-2	/	/
Methanol FEX		55.17	42.04 to 72.38
Ethyl acetate FEX		41.46	21.75 to 79.02
Acetone FEX		29.62	18.85 to 46.53

95% C.I.; 95% interval of confidence.

## Data Availability

All data can be obtained upon request from the corresponding author.

## Supplementary Materials

The following supporting information can be downloaded at: https://www.mdpi.com/article/10.3390/pharmaceutics15041291/s1, Supplementary Materials contains supplemental details on parts of the materials and methods section (when indicated in the main text), since parts of the experimental set-up were already described by our research group before. These details are summarized in Supplementary Materials to facilitate the readability of this article.
