# Supplementary material for: In Vitro Biotransformation and Anti-Inflammatory Activity of Constituents and Metabolites of Filipendula ulmaria
Source: Pharmaceutics. 2023 Apr 20;15(4):1291. doi: 10.3390/pharmaceutics15041291 (PMC10146082; doi:10.3390/pharmaceutics15041291)
Supplement: Supplementary file 1 [file pharmaceutics-15-01291-s001.zip › pharmaceutics-2311180-supplementary.pdf]

In Vitro Biotransformation and Anti-Inflammatory Activity of Constituents and Metabolites of *Filipendula ulmaria*

Anastasia Van der Auwera [1], Laura Peeters [1], Kenn Foubert [1], Stefano Piazza [2], Wim Vanden Berghe [3], Nina Hermans [1] and Luc Pieters [1,*]

[1] Natural Products & Food Research and Analysis (NatuRA), Department of Pharmaceutical Sciences, University of Antwerp, Universiteitsplein 1, 2610 Antwerp, Belgium; anastasia.vanderauwera@uantwerpen.be (A.V.d.A.); laura.peeters@uantwerpen.be (L.P.); kenn.foubert@uantwerpen.be (K.F.); nina.hermans@uantwerpen.be (N.H.)

[2] Laboratory of Pharmacognosy, Department of Pharmacological and Biomolecular Sciences, University of Milan, 20134 Milan, Italy; stefano.piazza@unimi.it

[3] Laboratory of Protein Chemistry, Proteomics & Epigenetic Signaling (PPES), Department of Biomedical Sciences, University of Antwerp, Universiteitsplein 1, 2610 Antwerp, Belgium; wim.vandenberghe@uantwerpen.be

* Correspondence: luc.pieters@uantwerpen.be

Supplementary Materials

S1. Preparation of Extract

A combination of extraction with H_2O:EtOAc and $CHCl_3$:MeOH:H_2O yielded both polar and apolar compounds. Approximately 18 g of sample was subsequently hydrated with 54 mL of water and 225 mL of EtOAc. The mixture was stirred for 2 h followed by 1 h of ultrasound-assisted extraction. Next, the solvent was removed, followed by washing the residue 3 times with 20 mL EtOAc and evaporating the extract to dryness. $CHCl_3$:MeOH:H_2O extraction was performed by adding 180 mL $CHCl_3$:MeOH:H_2O (4:4:2) to 18 g of sample. The mixture was stirred for 5 min, followed by 5 min of ultrasound-assisted extraction and stirred for an additional 15 min. The solvent was removed and the residue was washed 3 times with 20 mL $CHCl_3$:MeOH:H_2O (4:4:2). Finally, the dried H2O:EtOAc extract was redissolved with the extract from the $CHCl_3$:MeOH:H_2O extraction method, leading to an apolar and a polar phase. Both phases were separated, dried and stored in the dark at -20 °C.

S2. Gastrointestinal Biotransformation

Briefly, the pepsin solution was prepared by dissolving 16% (w/v) of pepsin powder in 0.1 M HCl (622 000 FIP-U/100 mL). To obtain the pancreatin-bile mixture, 0.4% (w/v) pancreatin and 2.5% (w/v) bile were dissolved in 0.1 M $NaHCO_3$ (32 000 FIP-U lipase, 143 600 FIP-U amylase, 16 400 FIP-U protease and 58.4 mmol bile/L). A fecal suspension of 10% (w/v) feces was prepared by homogenizing 3 human stool samples with a sterile phosphate buffer solution (0.1 M, pH 7.0) in sterile filter bags (Bagpage® R/25 400 mL, VWR International, Haasrode, Belgium) in a stomacher during 3 min in order to remove particulate food material. The phosphate buffer solution consists of $NaH_2PO_4.2H_2O$ (0.75% (w/v)), $Na_2HPO_4.2H_2O$ (1.03% (w/v)), sodium thioglycolate solution (3.45% (v/v)) and glycerol (17% (v/v)). The buffer solution was sonicated and autoclaved (1 bar, 121 °C). Prior to use, the 10% (w/v) fecal suspension was cultivated by adding 90% (v/v) phosphate buffer. After continuously stirring for 1 h at 37 °C the suspension is ready to use. In every step, fecal samples are processed in an anaerobic glove box (5% H2, 5% CO2 and 90% N2, Jacomex Glove Box T3, TCPS, Belgium) in order to ensure an anaerobic environment.

This biotransformation experiment included 3 groups: samples containing the polar *F. ulmaria* extract (treated with digestive enzymes and fecal microflora (FEX)), negative control samples (also containing

the extract with addition of digestive enzymes but not of fecal slurry (NCFEX)) and method blanks (containing no extract but comprising of an equal volume of solvent and undergoing treatment with digestive enzymes and fecal bacteria (MB)). For preparation of the FEX samples, an amount of approximately 300 mg of the polar *F. ulmaria* extract was weighed accurately in triplicate and mixed with 47 mL of ultrapure water. In the same manner, NCFEX samples were prepared in duplo (containing the same amount of *F. ulmaria* extract) and 3 MB samples comprising an equal volume of water.

Sample aliquots were taken at several time points during the experiment: before biotransformation (t0), after the gastric phase (S, 1 h), after the small intestinal phase (SI, 1.5 h) and during different time points of the colon phase (after 2, 4, 6, 10, 14, 18, 22, 24, 32, 40, 48 and 72 h). Samples were diluted with methanol (1:2) and centrifuged at 10 000 rpm for 10 min. The supernatant was diluted 10 times with MeOH:H2O (60:40) before analysis.

S3. Instrumental Analysis

For the qualitative UHPLC-UV-QTOF analysis of the obtained biotransformation samples, an aliquot of 5 µL was injected on a Waters Acquity UHPLC BEH SHIELD RP18 column (3.0 mm × 150 mm, 1.7 µm; Waters). The temperature of the column was kept at 40 °C. The mobile phase solvents consisted of water + 0.1% formic acid (A) and acetonitrile + 0.1% formic acid (B), and the gradient was set as follows: 0/2, 1/2, 14/26, 24/65, 26/100, 29/100, 31/2, 41/2 (min/B%). The flow rate was set at 0.4 mL/min. Accurate mass measurements were done using a Xevo G2-XS QTOF spectrometer (Waters, Milford, MA, USA) coupled with an ACQUITY LC system equipped with MassLynx 4.1 software. During the first analysis, full scan data were recorded in ESI (+) and ESI (−) mode from m/z 50 to 2000, and the analyzer was used in sensitivity mode (approximate resolution of 22 000 FWHM). The spray voltage was set at either +1.5 kV and −1.0 kV; cone gas flow and desolvation gas flow at 50.0 L/h and 1000.0 L/h respectively; source temperature and desolvation temperature at 120 °C and 500 °C respectively. Data were also recorded using MSE in positive and negative ionization modes, providing separate MS and MSE data. A ramp collision energy from 20 V to 30 V was applied to obtain additional structural information. Leucine-encephalin was used as lock mass. To monitor analytical drift and assess precision, quality control (QC) samples were injected after every time point. QC samples were prepared using a dilution of the standard solution mix (39 ng/mL).

S4. Data Analysis

In order to process the complex and dynamic data of the biotransformation experiment, a novel workflow was implemented to render as much information as possible from the longitudinal LC-MS data and to select the most interesting time profiles. Briefly, data were converted to open source .mzXML format to allow further processing. The XCMS CentWave algorithm was used to convert the raw data into features via peak-picking, followed by grouping, using following parameters: ppm = 10, peakwidth = c(5, 25), snthresh = 10, noise = 1000, mzdiff = 0.01, prefilter = c(3, 5000), integrate = 1. EDGE was used for the extraction of significant differential profiles since XCMS has the ability to discover features that are different between groups, but lacks the power to take longitudinal data into account. An interactive Shiny app developed in R, called tinderesting, was used to rate the quality of a subset of the resulting features. These labeled features were used to train a random forest model for predicting experts response. The machine learning model provided a single score for each feature, which is referred to as the tinderesting score. This score allowed ranking of all features based on the difference over time between the three groups (sample, blank and negative control). The maximal score of 1 corresponds to the model labeling this feature as interesting, whereas the minimal score of 0 defines an uninteresting feature.
